# A CNN-based model to count the leaves of rosette plants (LC-Net)

**DOI:** 10.1038/s41598-024-51983-y

**Published:** 2024-01-17

**Authors:** Mainak Deb, Krishna Gopal Dhal, Arunita Das, Abdelazim G. Hussien, Laith Abualigah, Arpan Garai

**Affiliations:** 1 Wipro Technologies, Pune, Maharashtra India; 2 Department of Computer Science and Application, Midnapore College (Autonomous), Paschim Medinipur, West Bengal India; 3 Department of Computer and Information Science, Linköping University, Linköping, Sweden; 4 Faculty of Science, Fayoum University, Fayoum, Egypt; 5 MEU Research Unit, Middle East University, Amman, Jordan; 6 Applied Science Research Center, Applied Science Private University, Amman, 11931 Jordan; 7 Hourani Center for Applied Scientific Research, Al-Ahliyya Amman University, Amman, 19328 Jordan; 8 Computer Science Department, Al al-Bayt University, 25113, Mafraq, Jordan; 9 Artificial Intelligence and Sensing Technologies (AIST) Research Center, University of Tabuk, 71491, Tabuk, Saudi Arabia; 10 Department of Electrical and Computer Engineering, Lebanese American University, 13-5053, Byblos, Lebanon; 11 College of Engineering, Yuan Ze University, Taoyuan, Taiwan; 12 Department of Computer Science and Engineering, Indian Institute of Technology, Delhi, India

**Keywords:** Computer science, Information technology, Software

## Abstract

Plant image analysis is a significant tool for plant phenotyping. Image analysis has been used to assess plant trails, forecast plant growth, and offer geographical information about images. The area segmentation and counting of the leaf is a major component of plant phenotyping, which can be used to measure the growth of the plant. Therefore, this paper developed a convolutional neural network-based leaf counting model called LC-Net. The original plant image and segmented leaf parts are fed as input because the segmented leaf part provides additional information to the proposed LC-Net. The well-known SegNet model has been utilised to obtain segmented leaf parts because it outperforms four other popular Convolutional Neural Network (CNN) models, namely DeepLab V3+, Fast FCN with Pyramid Scene Parsing (PSP), U-Net, and Refine Net. The proposed LC-Net is compared to the other recent CNN-based leaf counting models over the combined Computer Vision Problems in Plant Phenotyping (CVPPP) and KOMATSUNA datasets. The subjective and numerical evaluations of the experimental results demonstrate the superiority of the LC-Net to other tested models.

## Introduction

Recently, plant phenomics has attracted the rising attention of researchers. There is a need to increase the capabilities of high-yielding plants to ensure our food security. Global agriculture has faced significant challenges, including the need for high-yielding plants that can adapt to future climates and identifying the specific feedstock crop for biofuel production^1^. The nutrients, the supply of carbon, and the other outer environmental factors affect the size of leaves as well as the growth of a plant^2^. Plant phenotyping can provide an understanding of plant genes, correlations between plants and the environment, plant trails, etc. as well as new technologies to increase the yield of plants to overcome those problems. Plant phenotyping is also useful for studying the growth of a plant, the yielding rate of a plant, and also the internal structure of a plant. The traditional laborious and costly plant phenotyping techniques have become a bottleneck in plant breeding techniques as well as in functional genomics^1^. These phenotyping bottlenecks have prevented us from understanding the correlation between expressed phenotypes, genetic factors, and the condition of the environment^3^.

The possibility of connecting heritable traits to genetic markers depends on precise phenotypic assessment^4^. Plant image analysis is also a valuable tool for plant phenotyping. Image analysis has been used to analyse the plant trails and predict the growth of the plant, as well as give spatial information about the image. But measuring the visual characteristics of a plant is very costly and needs a very detailed long-term investigation to continue the observation. As a result, an automated approach for resolving the problem must be established. As recent literature suggests, the deep-learning (DL)-based methods are one of the most contemporary artificial-intelligence (AI) techniques and are currently a crucial component of plant phenotyping. There is a survey on plant phenotyping using computer vision with DL^5^.

Leaf area segmentation is the key feature of plant phenotyping and can be used to analyze the growth of a plant^2^. However, the segmentation of the leaves has become challenging when the size of the leaves is tiny or a significant number of leaves are overlapped. Also, the angle of the images and the lighting effect on the leaves can affect the efficiency of leaf area segmentation. Another crucial factor for analysing plant growth in plant phenotyping is determining the leaf count of the plant^6^. It is also challenging and time-consuming process to determine the number of leaves. A comprehensive plant phenotyping method for camera-captured images contributes to the cost reduction and improvement of plant and agricultural production. As a result, numerous researchers are looking into plant phenotyping using images recorded by cameras.

Furthermore, current CNN-based methods show their efficient performance in accurate leaf count predictions. A CNN is a class of neural networks within the field of DL. CNNs are designed with one or more convolutional layers and are mostly used for tasks such as image processing, classification, segmentation, and analysis of auto-correlated data. CNNs are primarily employed for the purpose of extracting features from grid-like matrix datasets, particularly in the context of image analysis. The CNN architecture encompasses various levels, including the input layer, convolutional layer, activation layer, pooling layer, and completely linked layers. The convolutional layer is responsible for applying filters to the input image in order to extract relevant features. The activation function is then applied in an element-wise manner to modify the matrix values, specifically converting negative values to zero. Following this, the pooling layer is employed to downsample the image, thereby reducing computational requirements. Finally, the fully connected layer is used to make the ultimate prediction. The process by which the network acquires the most effective filters is achieved through the use of backpropagation and gradient descent.

Therefore, in this manuscript, a novel CNN-based model for leaf counting has been proposed called LC-Net. The proposed model takes segmented leaf parts as additional input for achieving better leaf counting accuracy. Hence, well-established SegNet^7^ is utilized as it performs better leaf segmentation compared to existing well-established CNN-based models, namely, DeepLab V3+^8^, U-Net^9^, Fast FCN with Pyramid Scene Parsing (PSP)^10^, and Refine Net^11^. Lastly, the proposed LC-Net has been compared with the other state-of-the-art leaf count models, and the performance of all tested leaf count models has been tested over the combined CVPPP and KOMATSUNA datasets. The qualitative and numerical results indicate that the proposed LC-Net outperforms the existing leaf counting results.

In a nutshell, the significant contributions of the proposed work are enlisted in the following:

- A novel CNN-based model to count the number of rosette plants’ leaves has been proposed which has been provided two inputs i.e., segmented output and the original image for better accuracy
- The proposed LC-Net model is tested on the merged version of the dataset of KOMATSUNA and CVPPP (annotated for the leaf counting task by the experts). It is seen that the proposed LC-Net model outperforms existing state-of-the-art techniques.
- The proposed model use a normalization layer that is used to filter the unwanted pixels present in the images. Application of this layer in the proposed LC-Net is discussed in detail in Sect. "CNN based leaf segmentation".

The remainder of this manuscript is structured as follows. Next, Sect. "Related work" summarises about the existing work in the related field. Thereafter, Sect. "Methodology" discusses the proposed technique. Furthermore, Sect. "Experimental results" presents the experimental investigation. Lastly, Sect. "Conclusion and future work" contains the final observations and concluding remarks.

### Related work

The past few years have witnessed successful research work in the domain of phenotyping of plants. In the recent past, several research papers have been published on this hot topic. For example, in Ref.^12^, Tu et al. developed a leaf counting network architecture based on YOLO V3. They considered the problem of leaf counting as an object detection problem. As a result, first they detected the leaves and drew the bounding boxes, and then they counted the number of boxes. The proposed model achieved -0.32 of difference in count (DiC), 0.48 of Absolute difference in count (AbsDiC), 0.80 of Mean Squared Error (MSE), and 64.00% of percentage agreement on the CVPPP dataset. In Ref.^13^, another deep-learning based model called Eff-U-Net++ for leaf segmentation and counting had been proposed by Bhagat et al. Eff-U-Net++ was an encoder-decoder-based network. It had applied EfficientNet-B4 as the encoder sub-net. The model achieved 0.11, 0.03, 0.12 DiC and 0.21, 0.38, 1.27 AbsDiC on the CVPPP^14^, MSU-PID^15^, and KOMATSUNA^16^ datasets, respectively.

In Ref.^17^, two novel DL approaches had been proposed, elaborated, and compared by Farjon et al. for visual leaf counting tasks. The first model fed input image in different resolutions into the network that had ResNet-50 as the backbone network to estimate features of leaves at multiple scales. Next, in order to get the final count, repetitive regression of leaf heat map got from the previous step. The second model counted the number of leaves by locating the centers of the detected leaves and finally aggregating them. They got 1.17 MSE and 43.7% of the percentage agreement on the CVPPP dataset^14^. Miao et al.^18^ proposed a dataset for maize leaf counting and also proposed two DL methods to count the maize leaves. The first model counted the leaves by regression, and the second model counted the leaves by detection. In Ref.^19^, Lu et al. came up with a better way to use DL to count the dense leaves and leaves that overlap in natural environments. The developed approach was used to detect the object by merging a space-to-depth module, an Atrous spatial pyramid pooling, and a convolutional block attention module into the network. The experimental results showed that the improved DL approach achieved 96% accuracy. To improve the phenotyping process, in Ref.^20^, Karthik et al. introduced a unique semantic segmentation pipeline for the segmentation task. In CVPPP14 competition, a number of deep CNN models such as U-Net, Attention-Augmented Net, and Attention-Net were introduced. These networks were trained using the Arabidopsis Thaliana plant dataset. The Attention-Net achieved a 0.985 dice score, which is the best among others.

In Ref.^21^, Kumar et al. came up with a new orthogonal transform domain-based method to segment the leaf region and further counted it by fine-tuned deep CNN models. On the CVPPP dataset, fine-tuned AlexNet and VGG19 had been used to count the leaves and got 25.51%, 33.67% of percentage agreement, 5.43, 2.03 of MSE, 1.71, 1.03 of AbsDiC, and 0.39, 0.11 of DiC, respectively. In Ref.^22^, Buzzy et al. proposed a novel real-time object detection approach for the identification, localization, and quantification of plant leaves. A comparative analysis was conducted between a Tiny-YOLOv3 model and a faster R-CNN model. The Tiny-YOLOv3 and Faster R-CNN models were evaluated based on several performance metrics, including DiC, AbsDiC, MSE, and percentage agreement. The obtained values for these metrics were 0.25 and 0.0556, 0.8056 and 1.2778, 2.0833 and 2.8889, and 56% and 27.78%, respectively. Hati et al.^23^ presented a regression model for the purpose of leaf counting. The images were subjected to segmentation and subsequent enhancement, resulting in the removal of noise and transformation of the pixel data associated with the leaves. Subsequently, the images were inputted into the regression model, which is founded upon the architecture of AlexNet. In Ref.^24^, Ayalew et al. presented a domain-adversarial learning method in which a domain adaption technique was used to estimate a density map for leaf counting. Due to its flexibility in accommodating variations in distribution across source and destination datasets, the method exhibits potential for application in a broader spectrum of leaf counting and plant organ counting scenarios. The method got -0.95 of DiC, 1.56 of AbsDiC, 5.26 of MSE, and 29.33% of percentage agreement on the CVPPP dataset. Gomes and Zheng^25^ presented an experimental study on the limitations of datasets used for phenotyping and the performance strategy of the leaf segmentation tasks. They also looked at how test-time augmentation and model cardinality might help with single-class image segmentation. Another investigation had been conducted in Ref.^26^ by Yang et al. where they utilized a mask R-CNN based model to effectively segregate and classify leaf images that contained intricate backgrounds.

In the year 2019, a new approach^27^ to extract leaf regions from images of plants and count the number of leaves had been introduced by Kumar et al. There were three phases to the proposed methodology which are statistical image enhancing technique, a graph-based leaf area extraction approach, and a circular hough transform (CHT) based technique. In Ref.^28^, Valente et al. presented a preliminary study showcasing the efficacy of a trained deep neural network in accurately quantifying the number of leaves in plant images obtained by greenhouse workers through the use of handheld equipment. They got 0.31 of DiC, 0.62 of AbsDiC, 0.77 of MSE, and 47% of percentage Agreement. A Google Inception Net V3 based CNN model had been used by Jiang et al. in Ref.^29^ to count the number of maize leaves. To reduce redundant information, the Fisher Vector (FV) was used, and the Random Forest (RF) method was used to get the final prediction. They got 0.0018 of DiC, 0.35 of AbsDiC, and 0.31 of MSE.

In 2018, Giuffrida et al. presented a single deep network^30^ that counted the number of leaves from multi-modal 2D images of different species for any rosette-shaped plant. The model had a DiC of 0.19, an AbsDiC of 0.91, a MSE of 1.56, and a 32.9 percentage agreement. For the CVPPP dataset, the proposed approach achieved a segmentation accuracy of 95.4%, a DiC of 0.7 and an AbsDiC of 2.3. In Ref.^31^, Ubbens et al. proposed a plant phenotyping dataset by demonstrating that performance could be improved on the leaf counting task using 3D synthetic plants. These synthetic plants were also applied to augment a dataset. They recreated the architecture used in the reference experiment using the Ara2013-Canon dataset in the augmentation experiment^3^ for the augmentation experiment. They also switched the real and synthetic image datasets utilized to both train and test the DL-based model. Additionally, it was demonstrated that these datasets could be utilised in a comparable manner for training a neural network to accurately quantify the number of leaves.

For the plant phenotyping task, Aich et al. developed a data-driven strategy in Ref.^32^ that could be employed to a variety of plant species and imaging configurations. They used a deconvolutional network to segment the leaves, and the predicted images were used in a CNN for leaf counting. The model got 0.73 of DiC, 1.62 of AbsDiC, 4.31 of MSE, and 24.0 of Percentage Agreement on the CVPPP dataset. A Data Augmentation technique had been proposed by Kuznichov et al. in Ref.^33^ for segmenting the leaves followed by the counting the leaves from the images of rosette plants. In Ref.^34^, Itzhaky et al. developed two novel deep learning algorithms designed for the purpose of leaf item counting. The researchers utilised the CVPPP 2017 Leaf Counting Challenge dataset to demonstrate the efficacy of these methods. The findings show that they defeated the CVPPP challenge winner from 2017. In Ref.^35^, Pape and Kulkas introduced a methodology for leaf segmentation that utilises edge detection techniques. Additionally, they devised a method to analyse images by employing the software IAP^36^ in order to extract a multitude of image attributes that might be utilised for estimating the quantity of leaves.

The aforementioned discourse unequivocally illustrates the immense utility of performing precise tasks, such as leaf segmentation and counting, in the field of plant phenotyping. Furthermore, it should be noted that there exists a restricted range of CNN models and techniques that have demonstrated the capability to generate precise outcomes across two widely utilised datasets, namely CVPPP and KOMATSUNA. It has been observed that these algorithms frequently encounter difficulties in scenarios including a green background and/or a substantial amount of overlapping leaves. Consequently, in order to address this deficiency, the primary objective of this research endeavour is to construct a Convolutional Neural Network (CNN) model, specifically referred to as LC-Net. The LC-Net, as proposed, demonstrates the ability to achieve higher predictions in leaf counting even when confronted with various types of backgrounds. Furthermore, LC-Net demonstrates the capability to precisely quantify the number of leaves, even in cases when they are overlapping. The next part provides a detailed description of the LC-Net model that has been proposed, as well as an overview of the dataset that was utilised in this study.

## Methodology

The proposed LC-Net has been designed to count the number of leaves in the rosette plants. The suggested model takes both the RGB image of a rosette plant and the segmented image of its leaves as input in order to enhance accuracy. The subsequent subsections provide an exposition of the LC-Net architecture and an elucidation of the design’s underlying reasoning.

### CNN based leaf segmentation

The necessity of accurate leaf segmentation arises from the fact that the segmented leaf portion provides an additional benefit to the proposed LC-Net model, as detailed in Section 2.2. This study employs five well-known CNN models, namely DeepLab V3+ V3++^8^, SegNet^7^, Fast FCN with PSP^10^, U-Net^9^, and Refine Net^11^, to accurately segment the leaves of rosette plants. The effectiveness of an image segmentation CNN model is determined by its backbone. The backbone network is used to estimate the feature maps from the image, and followed by, these feature maps are further applied and processed in order to get desired result^37^. The backbone of the DeepLab V3+ model is the modified Aligned Xception^8,38^. VGG-16^39^ serves as the backbone for SegNet, while ResNet-101^40^ is utilized as the backbone for both FCN with PSP and Refine Net. U-Net^9^ segments using its own backbone. This paper adopts SegNet as the segmentation model for the proposed LC-Net because SegNet provides the best visually and numerically segmented results. In the section on experimental results, the segmented results of the tested CNN models are presented.

#### Normalization layer

**Figure 1:**
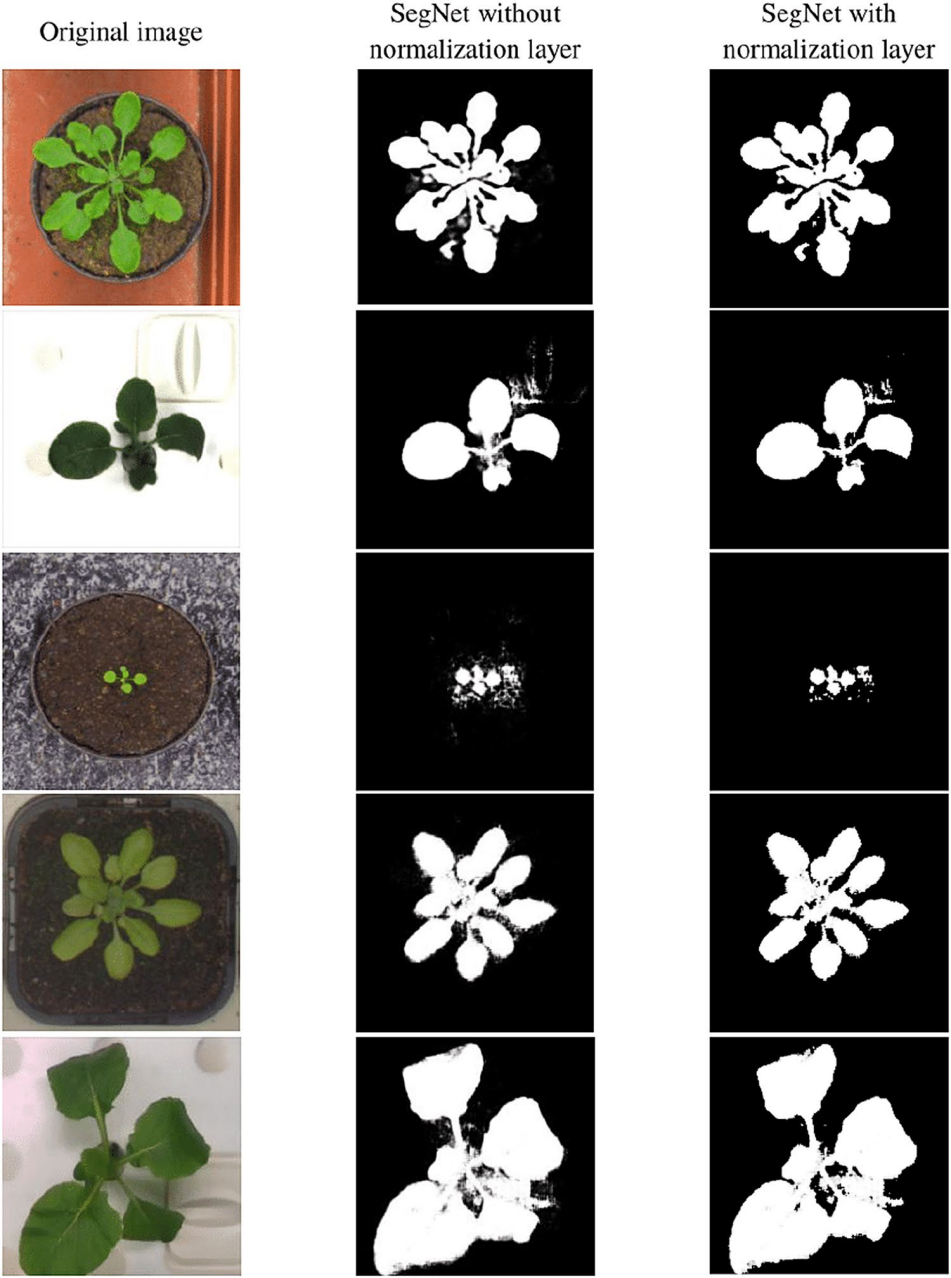
Visual representation of the significance of the normalization layer.

The normalization layer has been utilized to improve the segmented outcomes. This normalization Layer’s purpose is to eliminate any unwanted pixels from the images. The uneven background or light reflection could be the cause of these unwanted pixels. The precision of segmentation and leaf count may be affected as a result of these circumstances. The inclusion of the normalization layer has facilitated the construction of models that exhibit enhanced predictive accuracy. Figure 1 shows the segmented outcomes of SegNet with and without using normalization layer. Visual analysis clearly shows that utilization of normalization layer over SegNet’s output enhances the results.

### Proposed LC-Net model for leaf counting

**Figure 2:**
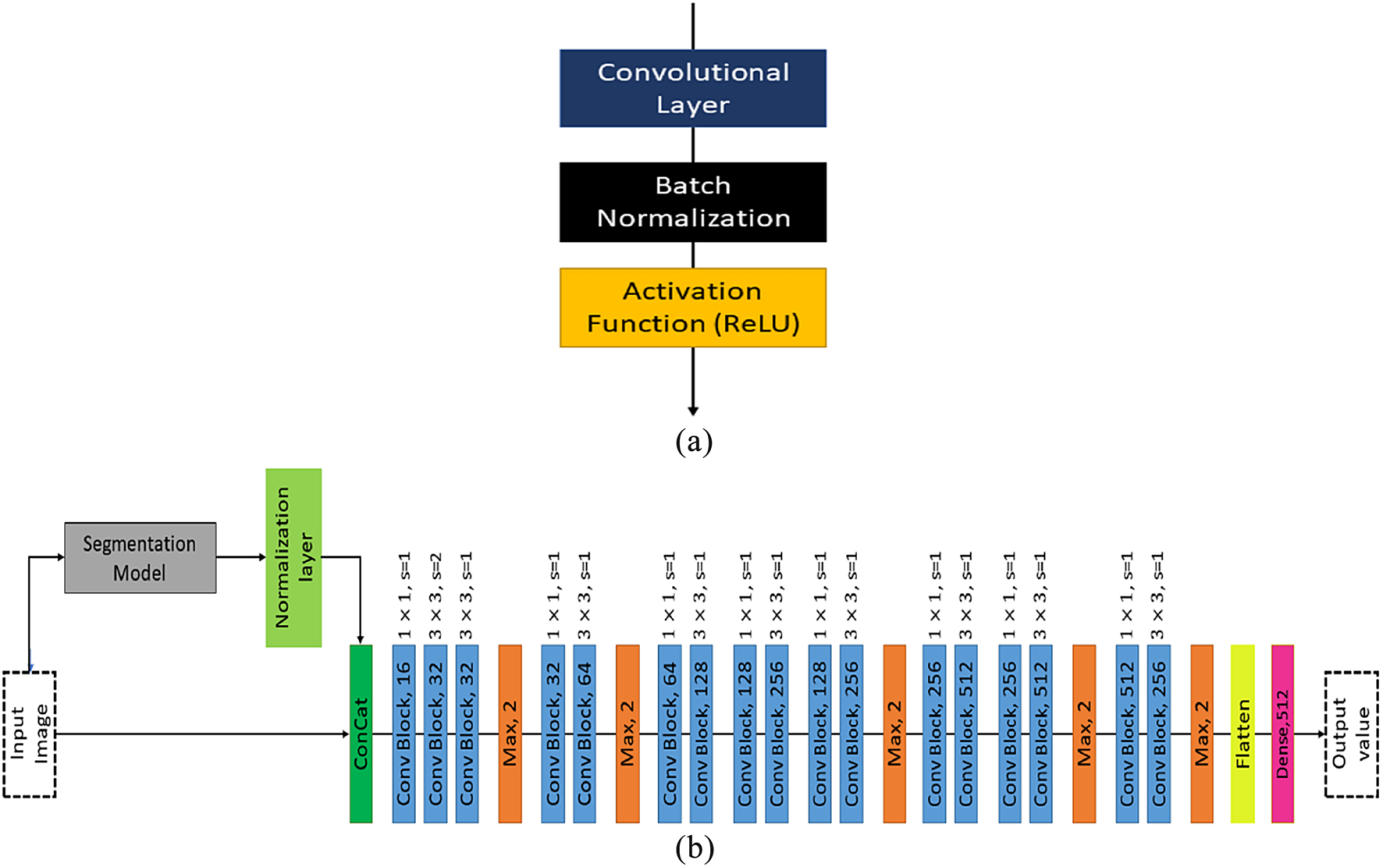
(**a**) Conv Block, (**b**) Architecture of the proposed LC-Net model.

This work adopts the conventional workflow of traditional computer vision systems in which the segmented output and counting model’s input are integrated as depicted in Fig. 2b which also indicates the entire architedture of the proposed model. The counting model has obtained additional information from the segmented output. Figure 2a indicates the structure of a Conv Block which is utilized in Fig. 2b. One Conv Block is made of convolution layer, batch normalization, and activation function. Batch normalization is a technique employed to enhance the speed and stability of the CNN. The leaf segmentation model and leaf counting model are trained independently of each other. The training of the counting model relies on the segmentation model, as the output generated by the segmentation model are utilised in conjunction with the RGB image input. Furthermore, the CNN models under consideration are trained without the inclusion of supplementary information pertaining to the specific species of the plant. LC-Net demonstrates a high level of accuracy in quantifying the quantity of leaves in a substantial portion of the dataset by solely utilising the segmented leaf regions as input. Nevertheless, it is worth noting that in certain instances, the segmentation model may generate inaccurate segmentations for a subset of the data. Consequently, it has the potential to impact the precision of leaf counting in the proposed LC-Net. Furthermore, the quality and texture of the original images are significant factors in CNN based leaf counting models. It has been observed within the dataset employed that certain images exhibit suboptimal quality, particularly in relation to leaf area. The average intensity of certain images is poor. Hence, the exclusive utilisation of original images also impacts the efficacy of the leaf counting model being proposed. As a consequence, both the original and segmented outcomes are inputted into the LC-Net in order to enhance the accuracy of leaf counting. We have also used a Normalization layer, which is nothing but a filter which makes the pixel values zero which are less than a threshold which is set to 0.5 from the experience during experiment. The Normalization Layer is elaborated in the section Section 2.1. The two inputs are concatenated at the beginning of the model. Then we have used one $(1\times 1)$ Conv Block followed by three $(3\times 3)$ Conv Block which is a replacement of one $(5\times 5)$ Conv Block to reduce the parameter size. After that a maxpooling layer is added then again one $(1\times 1)$ Conv Block followed by three $(3\times 3)$ Conv Block. We have used $(1\times 1)$ convolution with less filter size before all $(3\times 3)$ convolutions to reduce the number of parameters used in the model.

## Experimental results

The model is trained and tested using a system containing an NVIDIA GeForce 1650 having cuDNN CUDA 10.0, a 256 GB SSD, 16 GB RAM, and an AMD Ryzen 5 3550H CPU. TensorFlow and Scikitlearn were used in all of the studies. The experimental results have two folds: one is leaf segmentation and other is leaf counting. Both the results are presented in the following sub-sections.

### Dataset design

**Figure 3:**
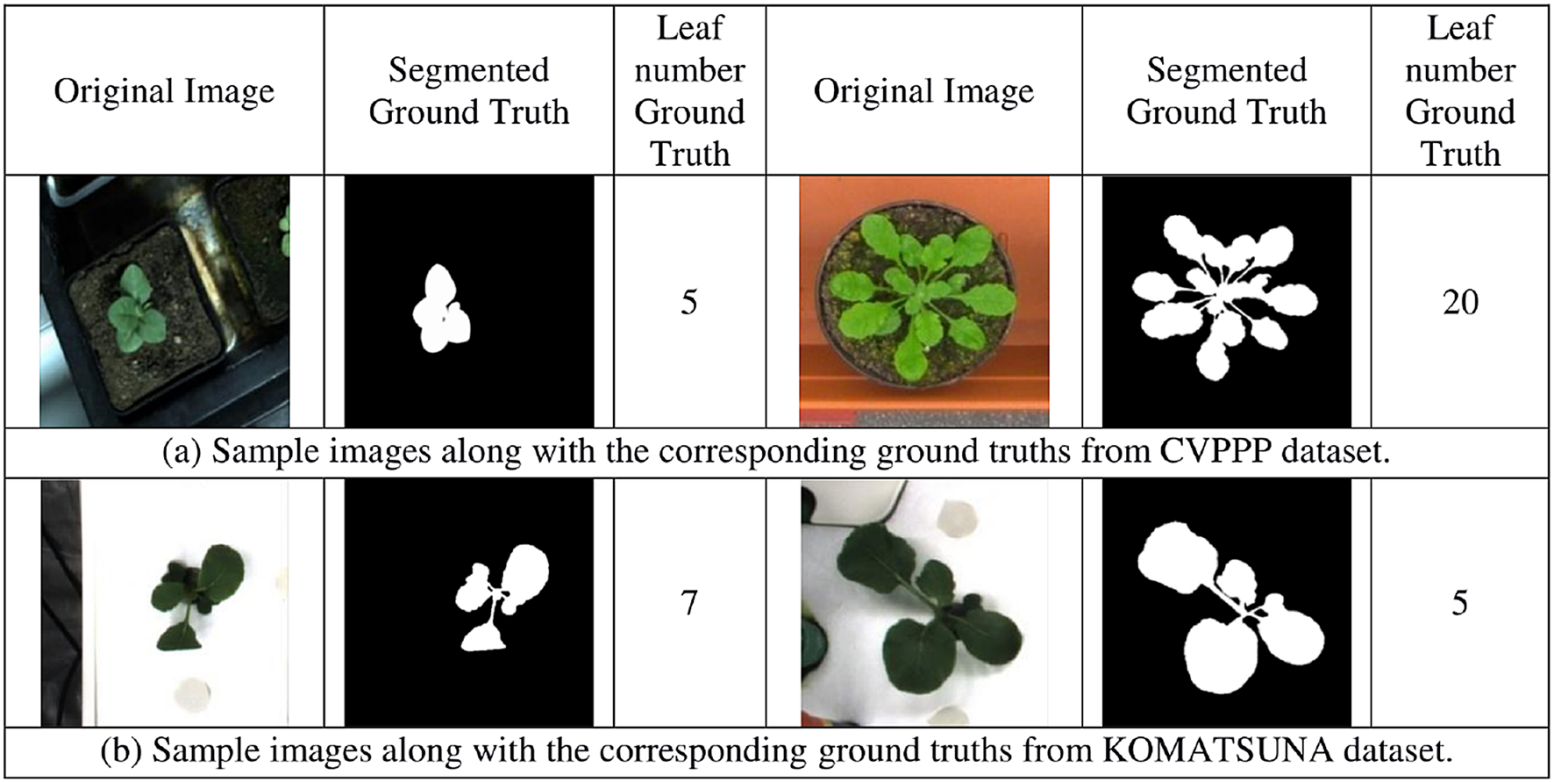
Samples of both utilized datasets.

As stated earlier, experiments on both the leaf segmentation and leaf counting have been done on two well-known datasets. They are (i) the KOMATSUNA [14] dataset, and (ii) the plant phenotyping benchmark dataset, known as CVPPP^14^. In our experiment, these datasets are merged. The KOMATSUNA dataset has been annotated for the leaf counting task by the experts. The CVPPP dataset have four sections. They are named as A1, A2, A3, and A4. All sections are included with segmented ground truth. On the other hand, there are two portions in the the KOMATSUNA dataset. Among them, one portion is made by capturing the images by a RGB-D camera, while the images in the other segment of the dataset is captured by several RGB cameras. As a whole, there are total of 1200 images in these two categories. As a result, there are a total of 2010 images along with the corresponding segmentation and leaf number ground truths in the the combined dataset. Bilinear interpolation^41^ has been used to resize the images into 224$\times$224. Subsequently, the combined dataset is partitioned into distinct subsets, namely the training, validation, and testing sets of images. In our experiment, the training set consists of 1410 images, whereas the validation and test sets each contain 300 images. In addition, a series of vertical flips, 90$^\circ$ and 180$^\circ$ clockwise and anticlockwise rotations are randomly applied in order to generate a greater quantity of images. In Fig. 3, some of the samples from each of the dataset are shown. In order to compare more accurately with state-of-the-art CNN models, another dataset is prepared using 160 images chosen randomly from the CVPPP dataset.

### Results of leaf segmentation

This section presents the results of CNN based leaf segmentation models. The five common CNN models such as Fast FCN with Pyramid Scene Parsing (PSP), DeepLab V3+, SegNet, U-Net, and Refine Net are employed for the proper leaf segmentation. For all CNN based leaf segmentation models, the well known ‘binary cross-entropy’ has been used as the loss function. In our experiment, the size of the input and output images are size 224 x 224 x 3 and 224 x 224 x 1, respectively. The batch size varies depending on the machine capacity and the architecture of the applied model. The suggested and existing tested CNN-based models’ parameter settings are provided in Table 1.

**Table 1:** Parameters Setting of the existing CNN-based models used for leaf segmentation.

CNN model	Parameter setting
DeepLab V3+	The input image has three channels and is (224, 224) in size. The backbone’s filters are identical to the original^8^. For prediction, the sigmoid activation function is utilised. DeepLab V3+ has 3.5M trainable parameters.
SegNet	The input image has three channels and is (224, 224) in size. The backbone’s filters are identical to the original^7^. For prediction, the sigmoid activation function is utilised. We have 16.7M trainable parameters for the SegNet model.
U-Net	The input image has three channels and is (224, 224) in size. The backbone and decoder filters are identical to the original^9^. Similar to the Fast FCN with PSP model, at the final level, the sigmoid activation function is applied in order to predict the class levels of each pixel. There are 31.0M trainable parameter present in the U-Net model used for the leaf segmentation.
Refine Net	The input image has three channels and is (224, 224) in size. The backbone and decoder filters are identical to the original^11^. Similar to the above mentioned models, at the last layer, the final prediction is performed using the sigmoid activation function. The Refine Net model have 89.1M parameters that are needed to be trained.

In order to evaluate and compare the performance of the segmentation process by the CNN models, three commonly used metrics such as, (a) segmentation accuracy (AC); (b) intersection over union (IoU); and (c) dice score (DI), are used. The quality metrics are summarised in Table 2.

**Table 2:** The quality metrics for leaf segmentation.

Sl.	Parameters	Remarks
1	AC	AC is the ratio of the total of correctly identified pixels and the total number of Pixels. The higher accuracy value represents better results^42^.
2	IoU	The value of IoU ranges between 0 and 1. It indicates the amount of overlapping is present between predicted image and the ground truth. In the existing literature, if the IoU for the outputs of the model is more than 0.5, then it is considered that the model is predicting well^43^.
3	DI	Basically DI indicates the similarity of predicted image with the ground truth. It is calculated by matching the overlapped region of the predicted image by the technique and the ground truth image^44^. A higher DI value better performance of the participating technique.

**Figure 4:**
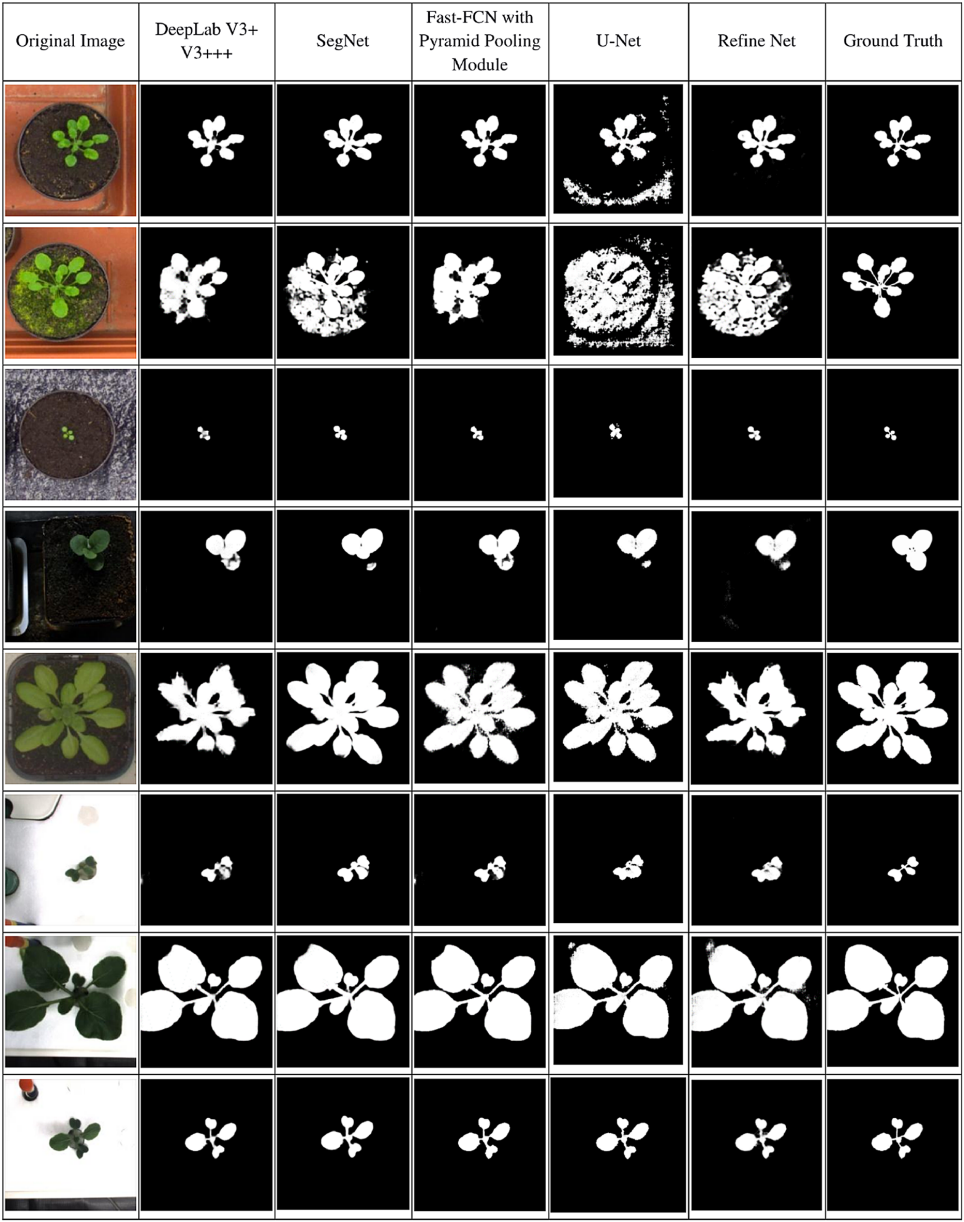
Qualitative comparison of the segmentation of proposed method with existing approaches.

In the training period, DeepLab V3+ shows the best performance among all. DeepLab V3+ achieves 97.38% of train accuracy, 97.30% of test accuracy, and 0.0108 of train loss. But while the models have been tested on the merged dataset and on the CVPPP dataset, the SegNet model shows the best results. Test results on the merged and CVPPP datasets are shown in Tables 3 and 4, respectively. SegNet achieves a 95.04% dice score and a 90.58% IoU. Therefore, the SegNet model has been chosen as the segmentation model for the proposed leaf counting model, i.e., LC-Net. The outcomes of segmentation by the existing tested CNN-based models along with the ground truth on the merged dataset are depicted in Fig. 4. From Fig. 4, it is quite clear that the SegNet model is performing with a significant accuracy.

**Table 3:** Test result that are obtained by various architectures on merged dataset (Test set size = 300 images).

Models	Test loss $(\times 10^{-3})$	Test accuracy (%)	IoU	Dice score
DeepLab V3+	22.3	97.06	89.78	94.59
SegNet	**17.6**	**97.29**	**90.58**	**95.04**
Fast FCN with PSP	19.4	97.24	89.29	94.31
U-Net	58.0	96.40	86.50	91.87
Refine Net	222.0	97.10	89.23	94.27

Significant values are in bold.

**Table 4:** Test result that are obtained by various CNN model on CVPPP Dataset (Test set size = 160 images).

Models	Test loss $(\times 10^{-3})$	Test accuracy (%)	IoU	Dice score
DeepLab V3+	23.7	97.21	85.11	91.79
SegNet	**19.7**	**97.92**	**86.04**	**92.50**
Fast FCN with PSP	21.1	97.57	85.94	91.54
U-Net	59.8	96.92	81.55	89.23
Refine Net	24.2	97.06	84.50	91.20

Significant values are in bold.

### Results of leaf counting

This section presents the results of CNN based leaf counting models. The proposed LC-Net has been compared to VGG^21^, Alex Net^21^, and the model proposed by Ubbans et. al.^31^ over merged dataset. In addition to that the proposed LC-Net is also additionally compared to counting models developed by Ayalew et. al.^24^, Giuffrida et. al.^30^, and Aich et. al.^32^ over CVPPP dataset for better comparison. The parameters settings for the mentioned CNN models are performed as per Table 5. For both images i.e. original image and predicted image, the size is considered as (224, 224). The batch size varied based on machine capacity and the model used.

**Table 5:** Parameter settings of the CNN-based models for leaf counting.

CNN model	Parameter setting
VGG	The original input image has three channels and segmented mask has one channel. The size of both input image is (224, 224). For prediction, the Linear activation function is utilised. We have 45M trainable parameters for VGG model of Kumar et. al. and used loss function is half MSE and the optimizer is SGD with 0.0001 learning rate, 0.9 momentum (As mentioned in Kumar et. al.^21^)
Alex Net	The original input image has three channels and segmented mask has one channel. The size of both input image is (224, 224). For prediction, the Linear activation function is utilised. We have 53M trainable parameters for Alex Net model of Kumar et. al. and used loss function is half MSE and the optimizer is SGD with 0.0001 learning rate, 0.9 momentum (As mentioned in Kumar et. al.^21^).
Ubbans et. al.	The original input image has three channels and segmented mask has one channel. The size of both input image is (224, 224). For prediction, the Linear activation function is utilised. We have 45M trainable parameters for proposed model of Ubbans et. al. and the loss function is MSE (As mentioned in Ubbans et. al.^31^).
Proposed LC-Net	The original input image has three channels and segmented mask has one channel. The size of both input image is (224, 224). For training, smooth L1 loss function has been used in Adam optimizer. We have 5M trainable parameters for our proposed LC-Net.

Four commonly used metrics namely^28,21^, (a) Mean Square Error (MSE); (b) Abstract DiC; (c) $R^2$; and (d) Percentage Agreement are utilized to study the performance of Leaf counting models. The brief descriptions of the mentioned metrics are reported in Table 6.

**Table 6:** Quality parameters for leaf count.

Sl.	Parameters	Remarks
1	Mean Square Error (MSE)	It’s calculated by taking the mean of the squares of the errors - i.e., differences between the predicated leaf number and the ground truth. Lower value indicates better results.
2	Absolute DiC (Difference in count)	The calculation involves determining the average value of the absolute differences between the predicted leaf number and the actual leaf number. Lower value indicates better results.
3	Coefficient of determination ($R^2$)	It measures how well the predicted values match the ground truth. High value indicates better results.
4	Percentage agreement	It takes the percentage of how many times the predicted value is exactly same as ground truth. High value indicates better results.

After training, we have tested all models on our merged test dataset and the CVPPP dataset as well. We have used four evolution measures, i.e., Abs DiC, MSE, $R^2$, percentage Agreement (%) and testing loss. Table 7 shows the test results of the models on the merged dataset, and Table 8 shows the test results of the models on the CVPPP dataset. The test results clearly show that the proposed LC-Net model is better than all other tested models and methods on both datasets by a large margin.

**Table 7:** Testing result of leaf count have obtained by different architectures on merged dataset (Test set size = 300 images).

Models	Test loss$\downarrow$	Abs DiC$\downarrow$	MSE$\downarrow$	$R^{2}\uparrow$	Percentage agreement (%)$\uparrow$
VGG^21^	0.6163	0.717	1.2325	0.9348	50.31
Alex Net^21^	0.728	0.7563	1.456	0.944	48.75
Ubbans et. al.^3^	4.3088	0.7203	1.1691	0.9493	46.72
Proposed LC-Net	**0.2371**	**0.4313**	**0.5924**	**0.9726**	**63.75**

Significant values are in bold.

**Table 8:** Testing result of leaf count have obtained by different architectures on CVPPP dataset (Test set size = 160 images).

Models/Methods	Abs DiC$\downarrow$	MSE$\downarrow$	Percentage agreement (%)$\uparrow$
AlexNet^21^	1.71	5.43	25.51
VGG19^21^	1.03	2.03	33.67
Ayalew et. al.^24^	1.56	5.26	29.33
Giuffrida et. al.^30^	0.91	1.56	32.9
Aich et. al.^32^	1.62	4.31	24.0
Proposed LC-Net	**0.65**	**1.13**	**51.39**

Significant values are in bold.

### Effect of the combined input

One of the major features of the proposed LC-Net is the combined input i.e. original image and segmented image. Therefore this section discusses the influence of this combined input in the proposed LC-Net. As a result, to study the consequences of combined input, a performance comparative study has been made with and without combined input. Hence, two CNN models have been compared which are (i) LC-Net with combined input, and (ii) LC-Net with original image input. Table 9 shows the numerical results of the LC-Net with combined and LC-Net with original image as input over CVPPP datasets. It can be seen that when the segmented image is combined with the original image the performance of the LC-Net model has been enhanced to some great extent. Best outputs are highlighted in bold in the tables. The presence of the $\uparrow$ symbol following a measure signifies that a higher value of that metric indicates a more favourable outcome. In contrast, the presence of the sign $\downarrow$ following the metric denotes the opposite.

**Table 9:** Numerical result for measuring the effect of combined input over CVPPP dataset (Test set size = 160 images).

Models	Abs DiC $\downarrow$	MSE $\downarrow$	Percentage agreement (%) $\uparrow$
LC-Net with combined input	**0.65**	**1.13**	**51.39**
LC-Net with original image input	1.06	3.98	42.87

Significant values are in bold.

## Conclusion and future work

This paper proposed a CNN network based model called LC-Net for counting the number of leaves of the rosette plants. The proposed LC-Net is both trained and evaluated using the images of KOMATSUNA and CVPPP datasets. It is seen that LC-Net performed with a good accuracy and produced a competitive outcome with respect to the state-of-the-art leaf counting models. The main feature of the proposed LC-Net is that is takes original and segmented leaf images as combined input which make it more robust for leaf counting because the segmented leaf part provides additional information to the proposed LC-Net. SegNet is used for leaf segmentation for the proposed LC-Net because SegNet provides the best visually and numerically segmented results compared to the other peer models. However, the major limitation of the work is that the proposed model has not been evaluated for too noisy images and which can be a great future work. In addition to that, further experiment regarding network structure design and loss function design may lead to a more accurate and robust CNN-based network for leaf counting. Last but not the least, a 3D convolution models may be used to analyze 3D leaf image data and it may produce more significant results. In the future, Authors can use many optimization algorithms and embedded them in the network for better accuracy. These algorithms can be any algorithm such as Snake Optimizer (SO)^45^, Fick’s Law Algorithm (FLA)^46^, Jellyfish Search (JS)^47^, Dandelion Optimizer (DO)^48^, Aquila Optimizer^49–51^, Atom Search Optimization (ASO)^52^, Water Cycle Algorithm (WCA)^53^, Bald Eagle Search (BES)^54^, African Vultures Optimization Algorithm (AVOA)^55^, Archimedes Optimization Algorithm (AOA)^56^, Beluga Whale Optimization (BWO)^57^, Hunter Prey Optimization (HPO)^58^, INFO^59^, Supply Demand Optimizer^60,61^, Reptile Search Algorithm (RSA)^62^, Golden Jackle Optimization (GJO)^63^, and more.

## Data Availability

The datasets that support the findings of this study are publicly available. Link for CVPPP dataset is: http://www.plant-phenotyping.org/datasets. Link for KOMATSUNA dataset is: https://limu.ait.kyushu-u.ac.jp/ agri/komatsuna/.

## References

[1] Furbank RT, Tester M (2011). Phenomics-technologies to relieve the phenotyping bottleneck. Trends Plant Sci..

[2] Walter A, Schurr U (1999). The modular character of growth in Nicotiana tabacum plants under steady-state nutrition. J. Exp. Bot..

[3] Ubbens JR, Stavness I (2017). Deep plant phenomics: A deep learning platform for complex plant phenotyping tasks. Front. Plant Sci..

[4] Jin S (2018). Stem-leaf segmentation and phenotypic trait extraction of individual maize using terrestrial LiDAR data. IEEE Trans. Geosci. Remote Sens..

[5] Chandra, A. L., Desai, S. V., Guo, W., & Balasubramanian, V. N. Computer vision with deep learning for plant phenotyping in agriculture: A survey. Preprint at arXiv:2006.11391. (2020).

[6] Telfer A, Bollman KM, Poethig RS (1997). Phase change and the regulation of trichome distribution in Arabidopsis thaliana. Development.

[7] Badrinarayanan V, Kendall A, Cipolla R (2017). SegNet: A deep convolutional encoder-decoder architecture for image segmentation. IEEE Trans. Pattern Anal. Mach. Intell..

[8] Chen, L. C., Zhu, Y., Papandreou, G., Schroff, F., & Adam, H. Encoder-decoder with atrous separable convolution for semantic image segmentation. In Proceedings of the European conference on computer vision (ECCV) (pp. 801-818) (2018).

[9] Ronneberger O, Fischer P, Brox T, Ronneberger O, Fischer P, Brox T (2015). U-net: Convolutional networks for biomedical image segmentation. International Conference on Medical Image Computing and Computer-Assisted Intervention.

[10] Wu, H., Zhang, J., Huang, K., Liang, K., & Yu, Y. Fastfcn: Rethinking dilated convolution in the backbone for semantic segmentation. Preprint at arXiv:1903.11816. (2019).

[11] Lin, G., Milan, A., Shen, C., & Reid, I. Refinenet: Multi-path refinement networks for high-resolution semantic segmentation. In Proceedings of the IEEE conference on computer vision and pattern recognition (pp. 1925-1934) (2017).

[12] Tu, Y. L., Lin, W. Y., & Lin, Y. C. Toward automatic plant phenotyping: starting from leaf counting. Multimedia Tools and Applications, 1-15 (2022).

[13] Bhagat S, Kokare M, Haswani V, Hambarde P, Kamble R (2022). Eff-UNet++: A novel architecture for plant leaf segmentation and counting. Eco. Inform..

[14] Minervini M, Fischbach A, Scharr H, Tsaftaris SA (2016). Finely-grained annotated datasets for image-based plant phenotyping. Pattern Recogn. Lett..

[15] Cruz JA, Yin X, Liu X, Imran SM, Morris DD, Kramer DM, Chen J (2016). Multi-modality imagery database for plant phenotyping. Mach. Vis. Appl..

[16] Uchiyama, H., Sakurai, S., Mishima, M., Arita, D., Okayasu, T., Shimada, A., & Taniguchi, R. I. An easy-to-setup 3D phenotyping platform for KOMATSUNA dataset. In Proceedings of the IEEE International Conference on Computer Vision Workshops (pp. 2038-2045) (2017).

[17] Farjon G, Itzhaky Y, Khoroshevsky F, Bar-Hillel A (2021). Leaf counting: Fusing network components for improved accuracy. Front. Plant Sci..

[18] Miao C, Guo A, Thompson AM, Yang J, Ge Y, Schnable JC (2021). Automation of leaf counting in maize and sorghum using deep learning. Plant Phenome J..

[19] Lu S, Song Z, Chen W, Qian T, Zhang Y, Chen M, Li G (2021). Counting dense leaves under natural environments via an improved deep-learning-based object detection algorithm. Agriculture.

[20] Karthik P, Parashar M, Reka SS, Rajamani KT, Heinrich MP (2022). Semantic segmentation for plant phenotyping using advanced deep learning pipelines. Multimedia Tools Appl..

[21] Kumar JP, Domnic S (2020). Rosette plant segmentation with leaf count using orthogonal transform and deep convolutional neural network. Mach. Vis. Appl..

[22] Buzzy M, Thesma V, Davoodi M, MohammadpourVelni J (2020). Real-time plant leaf counting using deep object detection networks. Sensors.

[23] Hati AJ, Singh RR (2021). Smart indoor farms: Leveraging technological advancements to power a sustainable agricultural revolution. AgriEngineering.

[24] Ayalew TW, Ubbens JR, Stavness I, Ayalew TW, Ubbens JR, Stavness I (2020). Unsupervised domain adaptation for plant organ counting. European Conference on Computer Vision.

[25] Gomes, D. P. S., & Zheng, L. Leaf Segmentation and Counting with Deep Learning: on Model Certainty, Test-Time Augmentation, Trade-Offs. Preprint at arXiv:2012.11486. (2020).

[26] Yang K, Zhong W, Li F (2020). Leaf segmentation and classification with a complicated background using deep learning. Agronomy.

[27] Kumar JP, Domnic S (2019). Image based leaf segmentation and counting in rosette plants. Inform. Process. Agric..

[28] Valente, J., & Giuffrida, M. V. Leaf counting from uncontrolled acquired images from greenhouse workers. Proceedings of the Computer Vision Problems in Plant Phenotyping (CVPPP 2019), Long Beach, CA, USA, 17. (2019).

[29] Jiang B, Wang P, Zhuang S, Li M, Li Z, Gong Z (2019). Leaf counting with multi-scale convolutional neural network features and fisher vector coding. Symmetry.

[30] Giuffrida MV, Doerner P, Tsaftaris SA (2018). Pheno-deep counter: A unified and versatile deep learning architecture for leaf counting. Plant J..

[31] Ubbens J, Cieslak M, Prusinkiewicz P, Stavness I (2018). The use of plant models in deep learning: An application to leaf counting in rosette plants. Plant Methods.

[32] Aich, S., & Stavness, I. Leaf counting with deep convolutional and deconvolutional networks. In Proceedings of the IEEE international conference on computer vision workshops (pp. 2080-2089) (2017).

[33] Kuznichov, D., Zvirin, A., Honen, Y., & Kimmel, R. Data augmentation for leaf segmentation and counting tasks in rosette plants. In Proceedings of the IEEE/CVF conference on computer vision and pattern recognition workshops (pp. 0-0) (2019).

[34] Itzhaky, Y., Farjon, G., Khoroshevsky, F., Shpigler, A., & Bar-Hillel, A. Leaf counting: Multiple scale regression and detection using deep CNNs. In BMVC (p. 328) (2018).

[35] Pape, J. M., & Klukas, C. Utilizing machine learning approaches to improve the prediction of leaf counts and individual leaf segmentation of rosette plant images. Proceedings of the Computer Vision Problems in Plant Phenotyping (CVPPP), 1-12 (2015).

[36] Klukas C, Chen D, Pape JM (2014). Integrated analysis platform: An open-source information system for high-throughput plant phenotyping. Plant Physiol..

[37] Minaee, S., Boykov, Y. Y., Porikli, F., Plaza, A. J., Kehtarnavaz, N., & Terzopoulos, D. Image segmentation using deep learning: A survey. IEEE Transactions on Pattern Analysis and Machine Intelligence. (2021). 10.1109/TPAMI.2021.3059968 33596172

[38] Qi, H., Zhang, Z., Xiao, B., Hu, H., Cheng, B., Wei, Y., Dai, J. Deformable convolutional networks coco detection and segmentation challenge 2017 entry. ICCV COCO Challenge Workshop (2017).

[39] Simonyan, K., & Zisserman, A. Very deep convolutional networks for large-scale image recognition. Preprint at arXiv:1409.1556. (2014).

[40] He, K., Zhang, X., Ren, S., & Sun, J. Deep residual learning for image recognition. In Proceedings of the IEEE conference on computer vision and pattern recognition (pp. 770-778) (2016).

[41] Patel V, Mistree K (2013). A review on different image interpolation techniques for image enhancement. Int. J. Emerg. Technol. Adv. Eng..

[42] Khan A, Sohail A, Zahoora U, Qureshi AS (2020). A survey of the recent architectures of deep convolutional neural networks. Artif. Intell. Rev..

[43] Rahman MA, Wang Y, Rahman MA, Wang Y (2016). Optimizing intersection-over-union in deep neural networks for image segmentation. International Symposium on Visual Computing.

[44] Sudre, C. H., Li, W., Vercauteren, T., Ourselin, S., & Cardoso, M. J. Generalised dice overlap as a deep learning loss function for highly unbalanced segmentations. In Deep learning in medical image analysis and multimodal learning for clinical (2017). 10.1007/978-3-319-67558-9_28 PMC7610921 34104926

[45] Hashim FA, Hussien AG (2022). Snake Optimizer: A novel meta-heuristic optimization algorithm. Knowl.-Based Syst..

[46] Hashim FA, Mostafa RR, Hussien AG, Mirjalili S, Sallam KM (2023). Fick’s Law Algorithm: A physical law-based algorithm for numerical optimization. Knowl.-Based Syst..

[47] Hu G, Wang J, Li M, Hussien AG, Abbas M (2023). EJS: Multi-strategy enhanced jellyfish search algorithm for engineering applications. Mathematics.

[48] Hu G, Zheng Y, Abualigah L, Hussien AG (2023). DETDO: An adaptive hybrid dandelion optimizer for engineering optimization. Adv. Eng. Inform..

[49] Yu H, Jia H, Zhou J, Hussien A (2022). Enhanced Aquila optimizer algorithm for global optimization and constrained engineering problems. Math. Biosci. Eng..

[50] Sasmal, B., Hussien, A. G., Das, A., & Dhal, K. G. A Comprehensive Survey on Aquila Optimizer. Archives of Computational Methods in Engineering, 1-28 (2023). 10.1007/s11831-023-09945-6 PMC10245365 37359742

[51] Izci D, Ekinci S, Hussien AG (2023). An elite approach to re-design Aquila optimizer for efficient AFR system control. PLoS ONE.

[52] Izci D, Ekinci S, Hussien AG (2023). Effective PID controller design using a novel hybrid algorithm for high order systems. PLoS ONE.

[53] Hussien AG, Hashim FA, Qaddoura R, Abualigah L, Pop A (2022). An enhanced evaporation rate water-cycle algorithm for global optimization. Processes.

[54] Chhabra A, Hussien AG, Hashim FA (2023). Improved bald eagle search algorithm for global optimization and feature selection. Alex. Eng. J..

[55] Zheng R, Hussien AG, Qaddoura R, Jia H, Abualigah L, Wang S, Saber A (2023). A multi-strategy enhanced African vultures optimization algorithm for global optimization problems. J. Computat. Des. Eng..

[56] Hashim FA, Khurma RA, Albashish D, Amin M, Hussien AG (2023). Novel hybrid of AOA-BSA with double adaptive and random spare for global optimization and engineering problems. Alex. Eng. J..

[57] Hussien, A. G., Khurma, R. A., Alzaqebah, A., Amin, M., & Hashim, F. A. Novel memetic of beluga whale optimization with self-adaptive exploration-exploitation balance for global optimization and engineering problems. Soft Computing, 1-39 (2023).

[58] Hassan, M. H., Daqaq, F., Kamel, S., Hussien, A. G., & Zawbaa, H. M. An enhanced hunter-prey optimization for optimal power flow with FACTS devices and wind power integration. IET Generation, Transmission & Distribution. (2023).

[59] Ekinci S, Izci D, Abualigah L, Hussien AG, Thanh CL, Khatir S (2023). Revolutionizing vehicle cruise control: An elite opposition-based pattern search mechanism augmented INFO algorithm for enhanced controller design. Int. J. Computat. Intell. Syst..

[60] Daqaq F, Hassan MH, Kamel S, Hussien AG (2023). A leader supply-demand-based optimization for large scale optimal power flow problem considering renewable energy generations. Sci. Rep..

[61] Hassan, M. H., Kamel, S., Shaikh, M. S., Alquthami, T., & Hussien, A. G. Supply-demand optimizer for economic emission dispatch incorporating price penalty factor and variable load demand levels. IET Generation, Transmission & Distribution. (2023).

[62] Sasmal, B., Hussien, A. G., Das, A., Dhal, K. G., & Saha, R. Reptile search algorithm: Theory, variants, applications, and performance evaluation. Archives of Computational Methods in Engineering, 1-29 (2023).

[63] Elseify MA, Hashim FA, Hussien AG, Kamel S (2024). Single and multi-objectives based on an improved golden jackal optimization algorithm for simultaneous integration of multiple capacitors and multi-type DGs in distribution systems. Appl. Energy.

